# Editorial: Community series in: the role of vitamin D as an immunomodulator, volume II

**DOI:** 10.3389/fimmu.2025.1733692

**Published:** 2025-11-13

**Authors:** Mourad Aribi, Franck J. D. Mennechet, Chafia Touil-Boukoffa

**Affiliations:** 1 Laboratory of Applied Molecular Biology and Immunology, University of Tlemcen, Tlemcen, Algeria, and Algerian Society for Experimental and Applied Immunology (ASEAI), Tlemcen, Algeria; 2 China-Algeria International Joint Laboratory on Emergency Medicine and Immunology, Guangdong Provincial, People’s Hospital (Guangdong Academy of Medical Sciences), Southern Medical University, Guangzhou, China; 3 Biotechnology Research Center (CRBt), Constantine, Algeria; 4 Institut National de la Santé et de la Recherche Médicale (INSERM) U1058, University of Montpellier, Montpellier, France; 5 Algerian Academy of Sciences and Technologies (AAST), Algiers, Algeria

**Keywords:** calcitriol (1,25(OH)_2_D_3_), immunomodulation, immune regulation, context-dependent immunity, innate and adaptive immunity modulation, metabolic disorders, immune-related diseases, vitamin D

## 1 Introduction

Vitamin D has emerged as a multifaceted molecule, acting not only as a regulator of metabolic and skeletal homeostasis but also as a modulator of immune responses (1–3). This dual functionality underscores the need to clarify the conceptual distinction between regulation and modulation, particularly within the framework of immunology. The following sections provide an integrated overview of the conceptual principles, molecular foundations, immunomodulatory mechanisms, and systemic relevance of vitamin D as a key homeostatic factor.

### 1.1 Molecular and physiological basis of vitamin D activity

Vitamin D continues to emerge as a pleiotropic regulator of immune and metabolic homeostasis, operating at the crossroads of endocrine, skeletal, and immune systems (4–6). Traditionally recognized for its role in calcium–phosphate balance and bone mineralization, vitamin D—functioning both as a vitamin and a pre-hormone—exerts wide-ranging biological effects through its active metabolite, 1,25-dihydroxyvitamin D_3_ [1,25(OH)_2_D_3_] (7–10). This metabolite binds to the vitamin D receptor (VDR), a ligand-dependent nuclear transcription factor expressed in numerous immune and non-immune cells, including those in the intestine, pancreas, bone, and skin (11). Upon activation, the 1,25(OH)_2_D_3_–VDR complex forms a heterodimer with the retinoid X receptor (RXR-α) and binds to vitamin D response elements (VDREs) in target genes, thereby regulating transcriptional programs involved in cell proliferation, differentiation, cytokine secretion, and immune tolerance (12–15).

Physiologically, vitamin D is synthesized in the skin from 7-dehydrocholesterol upon exposure to ultraviolet B radiation (290–320 nm) and subsequently hydroxylated in the liver and kidney by CYP2R1 and CYP27B1 to generate its active form (16, 17). The expression of these metabolic enzymes and the VDR within immune and non-immune tissues underscores the body’s intrinsic capacity to locally produce and respond to calcitriol, thereby modulating both autocrine and paracrine signaling networks. Beyond its classical skeletal actions, vitamin D plays critical roles in neuromuscular function and in regulating key cellular processes such as growth, differentiation, apoptosis, and glucose metabolism (18–25). Collectively, these multidimensional effects position vitamin D as a central integrator of immune regulation, metabolic balance, and overall systemic health.

### 1.2 Immunomodulation *versus* immune regulation: conceptual distinction

In immunology, the terms *immunomodulation* and *immune regulation* are often used interchangeably, yet they represent distinct biological concepts.

**Figure 1:**
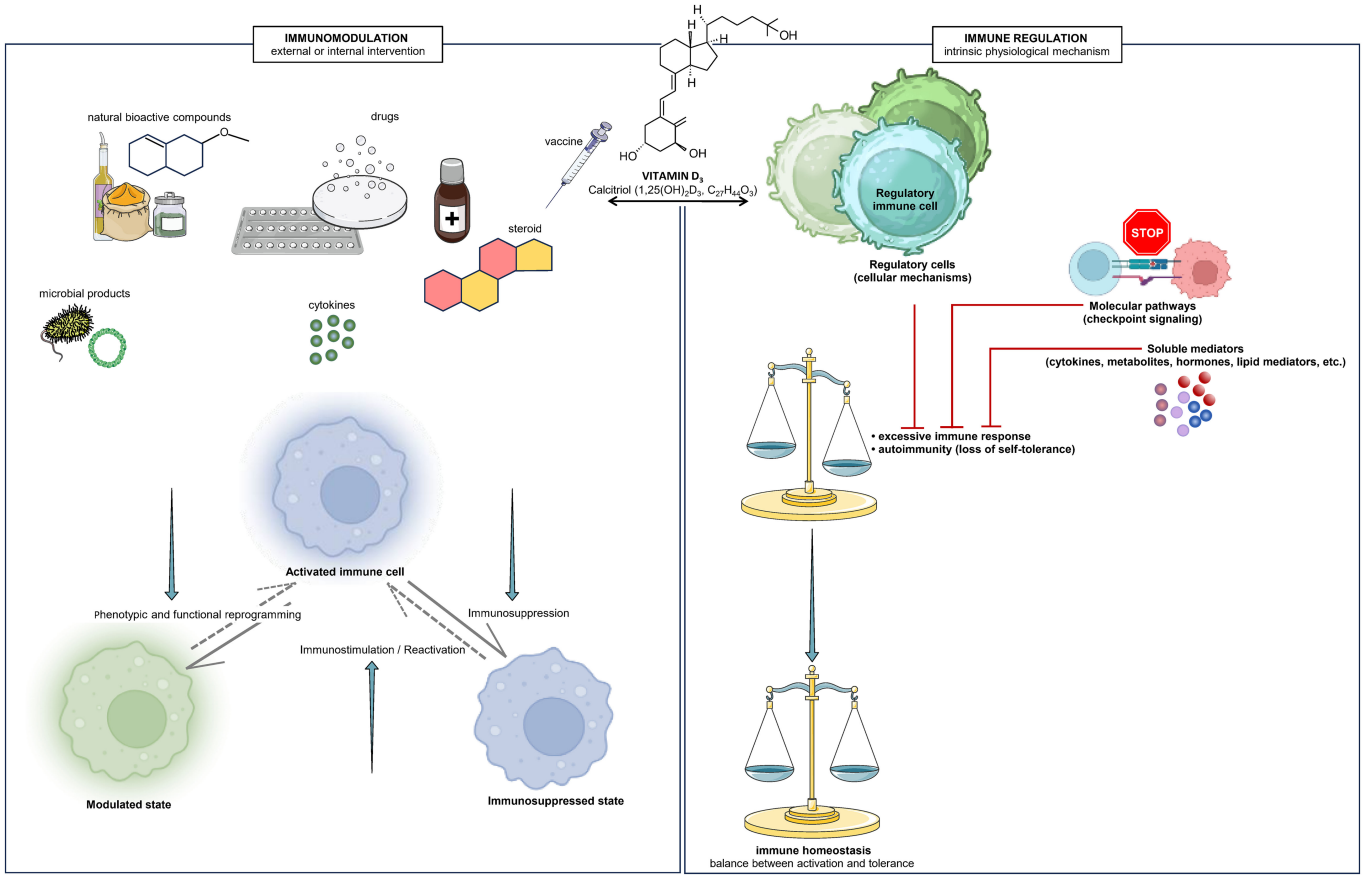
Conceptual interplay between immunomodulation and immune regulation: the dual influence of vitamin D. Immunomodulation refers to endogenous or exogenous influences that can bidirectionally modify immune responsiveness, either by stimulating, inhibiting, or redirecting cellular activities, thereby highlighting its adaptive and context-dependent nature. These processes affect immune cells that are already activated or primed, leading to a change in their functional and phenotypic state without complete loss of activation. In contrast, immune regulation represents intrinsic physiological mechanisms that maintain immune homeostasis and prevent both excessive immune activation and loss of self-tolerance. It reflects the immune system’s self-governing capacity to restore equilibrium following activation, through cellular mechanisms involving regulatory cells that directly suppress or modulate effector responses, molecular pathways engaging inhibitory checkpoint signaling that sustains immune homeostasis, and soluble mediators that limit inflammation and maintain immune tolerance. The bioactive form of vitamin D, 1,25-dihydroxyvitamin D_3_ (calcitriol), exemplifies a dual-acting molecule within this conceptual continuum. As a prohormone with immunomodulatory properties, it influences the differentiation, activation, and reprogramming of immune cells, while indirectly supporting immunoregulatory balance through its systemic endocrine and paracrine effects. All conceptual notions presented in this Figure are encompassed within this integrative framework. Schematic elements were partially adapted from Servier Medical Art (https://smart.servier.com), licensed under a Creative Commons Attribution 3.0 Unported License (CC BY 3.0). The molecular structure of the active form of vitamin D_3_ (calcitriol, 1,25(OH)_2_D_3_; C_27_H_44_O_3_) was generated using ACD/ChemSketch, Version 2.0 (Advanced Chemistry Development, Inc., Toronto, Canada).

Immune regulation refers to the intrinsic, homeostatic mechanisms by which the immune system maintains balance between activation and tolerance (26–28). These include key processes such as central and peripheral tolerance, the action of regulatory immune cells, cytokine feedback loops, and the suppression of excessive or self-reactive immune responses (29–31). In essence, immune regulation ensures the preservation of immune equilibrium and self-tolerance under physiological conditions. By contrast, immunomodulation encompasses any external or endogenous intervention—pharmacological, nutritional, hormonal, or microbial—that alters immune activity, either by enhancing or suppressing or redirecting specific cellular functions (32–34). Immunomodulators can therefore act as stimulants, suppressants, or balancing agents depending on the immunological context (**Figure 1**).

### 1.3 The dual and context-dependent immunomodulatory actions of vitamin D

Through interconnected pathways, vitamin D acts as a pivotal modulator of both innate and adaptive immunity. It strengthens antimicrobial defenses by inducing the expression of potent effector peptides, including cathelicidin and β-defensin 2, while simultaneously attenuating excessive inflammation by downregulating proinflammatory mediators (35–37). By fine-tuning cytokine networks and antigen-driven responses, vitamin D maintains a delicate equilibrium between immune activation and tolerance, promoting resolution of inflammation and preserving tissue integrity and homeostasis. Vitamin D thus exemplifies a potent immunomodulatory agent, as it influences both regulatory and effector immune mechanisms (38). It does not merely suppress or activate immune functions, but rather *modulates* their intensity and quality, promoting an adaptive state of functional equilibrium that is essential for immune homeostasis. In this regard, vitamin D serves as a paradigm of *context-dependent immunomodulation* (39)—a molecular sentinel that operates through finely regulated receptor-mediated signaling to maintain immune homeostasis.

## 2 The immunomodulatory power of vitamin D: key insights from volume II

The compelling body of work compiled here, comprising 14 original research and review articles, highlights the growing interest of vitamin D and significantly advances our understanding of its immunomodulatory effects across a broad spectrum of conditions—from allergic and autoimmune diseases to viral infections like COVID-19, pregnancy-related disorders, inflammatory bowel diseases, metabolic syndromes, and cancer.

This editorial synthesizes the key contributions of this volume, emphasizing the convergence of mechanistic insights and clinical applications.

### 2.1 UVB-driven vitamin D metabolism and macrophage reprogramming fine-tuned by physical shielding

The experimental study by Meterfi et al. reveals how environmental factors dynamically shape vitamin D-mediated immune regulation. By exposing peritoneal macrophages to UVB radiation with or without a simulated passive barrier, the authors demonstrate that UVB enhances respiratory burst, MPO expression, and M1-like polarization, while physical shielding fine-tunes these effects by attenuating METosis-related MPO activity and modulating local redox metabolism. This study highlights how physical environmental constraints can calibrate vitamin D metabolism and innate immune effector functions, providing mechanistic insight into how external cues orchestrate immune homeostasis.

### 2.2 Vitamin D in allergic diseases and immunotherapy

Allergic disease receives careful attention: the review by Zhang et al. synthesizes mechanistic and clinical evidence linking vitamin D to atopic dermatitis, allergic rhinitis and asthma. In a complementary piece, Zúñiga and Bazan-Perkins analyze the controversial relationship between vitamin D and atopy, highlighting that vitamin D promotes a pro-tolerogenic Th2 phenotype, suppresses Th1-driven inflammation, and supports immune homeostasis. They further emphasize that vitamin D acts through regulatory T-cells to maintain cytokine balance—modulating both pro- and anti-inflammatory pathways—and by limiting B-cell proliferation and differentiation, thereby fostering a state of immune equilibrium.

### 2.3 Pregnancy, hemostasis and vitamin D-associated platelet modulation

In obstetric immuno-hematology, the clinical study by Wang et al. shows that gestational hypertension is associated with lower vitamin D levels and platelet counts (PLT), along with higher mean platelet volume (MPV), platelet distribution width (PDW), and D-dimer levels. Vitamin D was significantly correlated with these parameters, suggesting a potential role in modulating platelet function and coagulation, and highlighting the value of optimizing vitamin D status in managing this condition.

### 2.4 Vitamin D and COVID-19 in risk modulation and clinical outcomes

The role of vitamin D during the COVID-19 pandemic is examined from multiple complementary angles. Missilmani et al. report that while systemic serum 25(OH)D levels alone are not directly associated with COVID-19 status, elevated nasopharyngeal VDR and DEFA1–3 expression correlate with reduced risk of SARS-CoV-2 infection, underscoring the importance of local vitamin D responsiveness. Complementing these findings, Petakh et al., in an umbrella review of systematic reviews, conclude that vitamin D supplementation significantly reduces the risk of Intensive Care Unit (ICU) admissions and mortality in COVID-19, particularly in vitamin D–deficient populations, while underscoring the need for standardized study designs. The cohort study by Konikowska et al. further establishes that sufficient vitamin D levels (≥30 ng/mL) are associated with improved survival and a reduced risk of death among hospitalized COVID-19 patients, highlighting the prognostic value of 25(OH)D concentrations during hospitalization. In pediatric settings, Perestiuk et al. demonstrate that vitamin D deficiency or insufficiency is associated with more severe acute disease, prolonged hospitalization, and a 2.2-fold higher risk of long COVID—particularly with neurological and musculoskeletal symptoms—underscoring the importance of age-specific preventive approaches.

### 2.5 Vitamin D status across metabolic and inflammatory disease outcomes

Population and clinical cohort studies add epidemiological weight to mechanistic findings. Liu et al. report that lower serum 25(OH)D is associated with a higher incidence of hyperlipidemia in adults, highlighting the link between lifestyle-related vitamin D insufficiency and adverse lipid profiles. In the inflammatory bowel disease field, Zheng et al. report that higher baseline vitamin D levels independently predict clinical remission in infliximab-treated Crohn’s disease patients, suggesting that vitamin D status may influence biologic therapy outcomes and patient stratification.

### 2.6 Vitamin D in autoimmunity: immunomodulation and genetic evidence

In their Opinion article, Su et al. revisit the debated field of high-dose vitamin D supplementation as a potential approach to immune recalibration in autoimmune diseases, highlighting its capacity to modulate Th1, Th17, and regulatory T-cells, alongside B-cell activity, in selected conditions, including multiple sclerosis, systemic lupus erythematosus, and Crohn’s disease. Adding a genetic perspective, Huang et al. applied a Mendelian randomization framework leveraging genetic variants linked to various circulating micronutrients, including vitamin D, to dissect causal relationships with systemic lupus erythematosus (SLE). Using summary statistics from the IEU OpenGWAS database and supported by multiple sensitivity analyses (including MR-PRESSO, MR-Egger, and leave-one-out), their findings provide genetic evidence for a protective role of selected nutrients—notably vitamin D and calcium—against autoimmune susceptibility, highlighting the value of integrative nutrigenomic approaches.

### 2.7 Mechanistic and nanotechnological advances in vitamin D-based cancer therapy

Two studies in this volume shed light on novel oncological applications of vitamin D. Zhang et al. reveal an immunosuppressive 1,25(OH)_2_D_3_–LL-37–tumor-associated macrophage (TAM) axis in hepatocellular carcinoma (HCC), in which LL-37 induction promotes macrophage recruitment and M2 polarization, thereby limiting vitamin D’s efficacy. Importantly, the authors show that suramin, an LL-37 inhibitor, disrupts TAM recruitment and M2 polarization, restores M1 polarization, inhibits protein kinase B/mammalian target of rapamycin (Akt/mTOR) and signal transducer and activator of transcription 3 (STAT3) signaling, and enhances 1,25(OH)_2_D_3_ therapeutic effects, reducing tumor growth and nodules *in vivo*, highlighting its potential as an adjunct in vitamin D–based immunotherapy for HCC.

To overcome the narrow therapeutic window of vitamin D_3_ in oncology, Ezcurra-Hualde et al. developed a liposomal cholecalciferol formulation (VD-LP) with high encapsulation efficiency and long-term stability. VD-LP modulated immune-related and metabolic gene expression in THP-1 cells and exerted superior antiproliferative effects across colorectal, breast, and prostate cancer cell lines compared to free vitamin D_3_. *In vivo*, VD-LP delayed tumor growth and improved survival without inducing hypercalcemia, highlighting its favorable toxicity profile. These findings demonstrate how nanotechnological innovations can safely extend vitamin D’s therapeutic applications in oncology field.

## 3 Concluding perspectives: mechanistic integration and emerging frontiers

Together, the studies featured in this volume illuminate how vitamin D acts as a central immunometabolic regulator, bridging molecular mechanisms with translational vision across diverse pathophysiological contexts. From redox-sensitive macrophage reprogramming and modulation of T helper (Th) cells to genetic and nanotechnological innovations, these contributions collectively underscore vitamin D’s capacity to fine-tune immune responses, metabolic signaling, and therapeutic efficacy.

The integration of multi-omic approaches, including genomics, transcriptomics, and nutrigenomics, emerges as a powerful avenue for uncovering individualized responses to vitamin D and identifying predictive biomarkers of efficacy across diseases.

Looking ahead, the convergence of precision nutrition, immune engineering, and vitamin D-based interventions define an exciting frontier for research, paving the way toward personalized immunomodulatory strategies that harmonize metabolic, genetic, and environmental dimensions of health.

In conclusion, this Research Topic provides a comprehensive framework for understanding and advancing vitamin D-based interventions across immune, metabolic, and oncological landscapes, charting a path toward more precise and effective clinical and therapeutic applications.

We trust that the insights presented here will catalyze translational breakthroughs and support the rational design of vitamin D-based interventions tailored to individual immune and metabolic profiles.
